# The Effect of Incorporating Lüders Plateau into a Plasticity Model in Predicting the Ballistic Impact Responses of 40CrNiMoA Steel Projectiles and Plates

**DOI:** 10.3390/ma18061364

**Published:** 2025-03-19

**Authors:** Yahui Shi, Xinke Xiao, Bin Jia, Yuge Wang, Jicheng Li

**Affiliations:** 1 School of Civil Engineering, Nanyang Institute of Technology, Nanyang 473004, China; shiyahui23@gscaep.ac.cn (Y.S.);; 2 Henan International Joint Laboratory of Dynamics of Impact and Disaster of Engineering Structures, Nanyang Institute of Technology, Nanyang 473004, China; 3 State Key Laboratory of Explosion Science and Safety Protection, Beijing Institute of Technology, Beijing 100081, China; 4 Institute of Systems Engineering, China Academy of Engineering Physics, Mianyang 621999, China

**Keywords:** 40CrNiMoA high-strength steel, Johnson–Cook plasticity model, Lüders plateau, ballistic impact tests, Taylor impact tests

## Abstract

Lüders plateau, a frequently observed phenomenon in uniaxial tensile tests of 40CrNiMoA high-strength steel, significantly influences material fracture behavior but is often neglected in characterizing metal plasticity and fracture properties. This study aims to develop a modified Johnson–Cook-2 (MJC-2) plasticity model incorporating Lüders plateau effects and evaluate its predictive capability for impact response. A series of mechanical tests were conducted and the plasticity model was calibrated through an experimental–numerical approach. Taylor impact and ballistic impact tests were conducted using a single-stage gas gun, with corresponding numerical simulations performed in finite element (FE) software. The results demonstrate that the MJC-2 model provides superior accuracy in predicting the fracture behavior of both targets and Taylor rods, as well as ballistic limit velocities (BLVs). Compared to models neglecting Lüders plateau, MJC-2 significantly enhances prediction precision.

## 1. Introduction

High-strength steel, known for its excellent strength-to-weight ratio, significantly enhances the ballistic resistance of military equipment and structures and has been widely used in military applications, such as armored vehicles, tanks, warships, etc., to resist the high-speed impact of bullets [1,2]. Projectile penetration often involves complex failure modes, along with high temperatures and strain rates and large strains [3]. Therefore, to accurately analyze impact issues, numerical simulation is usually an important approach, capable of simulating the impact process and monitoring the instantaneous stress, strain, and deformation of both projectiles and targets. Over the past few decades, a considerable amount of research has been conducted to investigate the ballistic impact behavior of metallic targets using a hybrid experimental–numerical approach [4,5,6,7,8,9,10]. Accordingly, a valuable conclusion was obtained that an accurate material model and reliable parameters are necessary for accurately reproducing the dynamic behavior of targets under impact loadings. Lin et al. [11] also pointed out that the accuracy of numerical simulations depends greatly on the accurate characterization of how the material model effects the material properties.

For certain metallic materials, Lüders plateau appears in the stress–strain curves immediately following the elastic stage, which is a stress discontinuity phenomenon associated with strain localization. This characteristic results from material instability caused by dislocations induced by nitrogen and carbon atmospheres, commonly observed in mild steels [12,13]. Previous studies have found that the existence of Lüders plateau may affect the ductile fracture behavior of steels [14,15]. Therefore, it is crucial to introduce Lüders plateau into the material model in simulating the dynamic fracture behavior of metallic materials.

The Johnson–Cook (JC) model [16,17] is most frequently used to characterize the plasticity and fracture behavior of metallic materials under dynamic loadings. Accordingly, the C model was calibrated to describe the deformation behavior of various metallic materials [18,19,20,21,22,23], in which Xiao et al. [24] modified the JC model by replacing the Ludwik equation [25] with a linear combination of the Ludwik equation and Voce’s law [26], and then the modified JC (MJC) model was used to establish the dynamic constitutive relationship of 2024-T351 aluminum alloy, and the modification was proven to be effective. Following this modification strategy, the deformation and failure behaviors of many metallic materials under dynamic loading conditions have been successfully predicted over the past few decades [27,28,29,30,31,32,33]. However, this MJC model did not take Lüders plateau into consideration. Therefore, in this paper, two parameters, $e_{0}$ and $C_{2}$, are further added to the MJC model to accurately capture Lüders plateau phenomenon, and it will be called the MJC-2 model.

**Figure 1:**
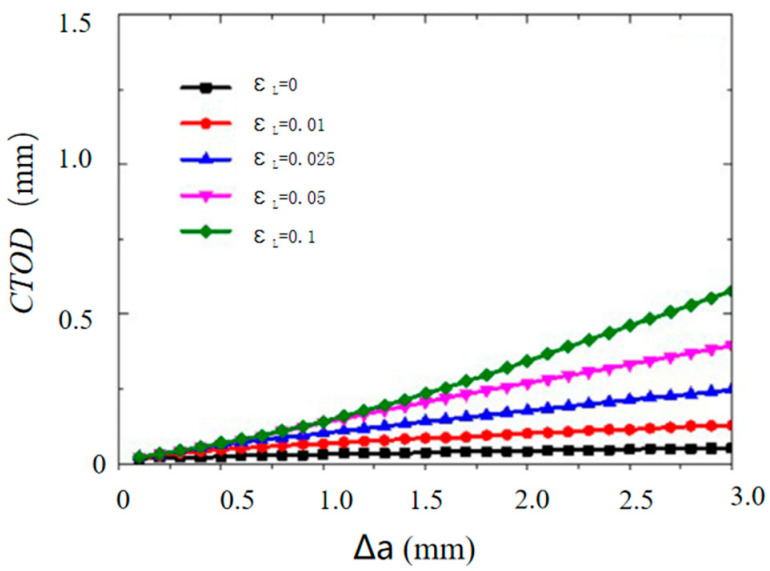
Impact of Lüders plateau on resistance curves for SENT specimens [34].

**Figure 2:**
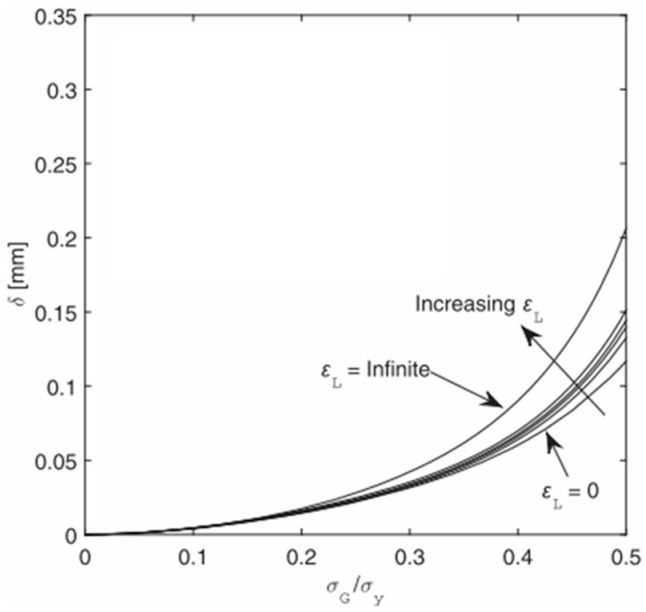
CTOD versus gross stress level for 0, 1%, 2%, 3%, 5%, and infinite Lüders strains, with yield strengths from 300 MPa to 800 MPa [35].

**Figure 3:**
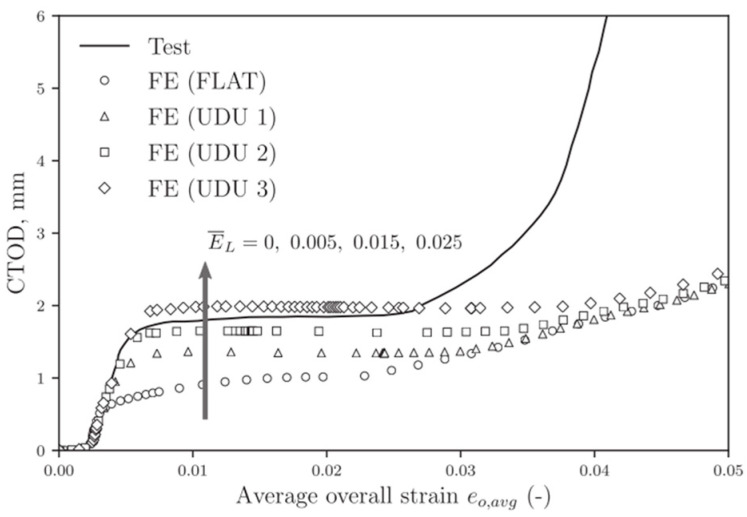
Comparison of CTOD for an average crack size of 4.41 × 100 mm from tests and FE analyses, excluding ductile tearing [37].

Over the past few decades, some numerical simulations have been carried out by introducing Lüders plateau into material models. Tu et al. [34] studied the influence of Lüders plateau on crack growth resistance using a cohesive zone model. They found that Lüders plateau has a minimal effect on crack growth resistance at low cohesive energy. However, as cohesive energy increases, the impact of Lüders plateau becomes more pronounced, with materials exhibiting longer Lüders plateaus demonstrating higher resistance curves, as presented in Figure 1. Dhal et al. [35] examined the impact of Lüders plateau on the crack driving force, measured as crack tip opening displacement, in single-edge-notched-tension specimens through FE simulation. They observed that Lüders plateau increased the crack driving force, with a larger plateau leading to a greater increment, as presented in Figure 2. Wang et al. [36] explored the fracture behavior of X65 pipes with circumferential defects in the presence of Lüders plateau. They determined that the up–down–up (UDU) model provided suitable accuracy and conservativeness for evaluating the fracture response of defective pipes exhibiting Lüders plateau. In contrast, existing stress- and strain-based fracture assessment methods often underestimate the crack driving force during the Lüders plateau stage, as presented in Figure 3. According to the literature review above, it is known that most of the numerical simulations considering Lüders plateau are limited to quasi-static loading, and few studies have established Lüders plateau-related plasticity models and fracture criteria to predict dynamic loading events, and the value of incorporating Lüders plateau into material models in predicting the ballistic impact behavior of metallic materials remain unclear.

In the present paper, the effect of incorporating Lüders plateau into a plasticity model predicting the ballistic impact behavior of 40CrNiMoA steel plates is investigated. In Section 2, the mechanical testing technique is introduced and the corresponding test results in terms of stress–strain relations and fracture morphologies are presented and discussed. In Section 3, the MJC-2 model considering Lüders plateau is established and then the MJC-2 model as well as the MJC model are calibrated by using a hybrid experimental–numerical approach. Section 4 and Section 5 introduce the ballistic impact and Taylor impact techniques, and the MJC-2 model and the MJC model are used to simulate the corresponding impact tests, and then the case with Lüders Plateau and the reference case without Lüders Plateau are compared with each other. In Section 6, the stress triaxiality and fracture strain of the failure zone are analyzed, and the influence of Lüders plateau on the numerical simulation of the ballistic impact behavior of 40CrNiMoA is discussed. Finally, the main findings are summarized in Section 7.

## 2. Mechanical Tests

### 2.1. Specimen Material

The raw material employed in this study is a 40 mm-diameter 40CrNiMoA high-strength steel rod. 40CrNiMoA represents a class of ultra-high-strength alloy structural steel, which is manufactured by strategically incorporating specific alloying elements into high-quality carbon structural steel. This advanced material exhibits an exceptional combination of mechanical properties, including superior strength, enhanced toughness, and excellent hardenability, making it particularly suitable for demanding structural applications. Its chemical composition is presented in Table 1.

Prior to processing, the material underwent a quenching and tempering heat treatment process. The thermal treatment protocol consisted of austenitizing at 850 °C for 30 min followed by oil quenching, with it subsequently tempered at 650 °C for 150 min with water cooling. This optimized heat treatment regimen resulted in a homogeneous microstructure within the 40CrNiMoA steel, yielding superior mechanical properties that are essential for high-performance applications.

### 2.2. Quasi-Static Tensile Tests at Room Temperature

**Figure 4:**
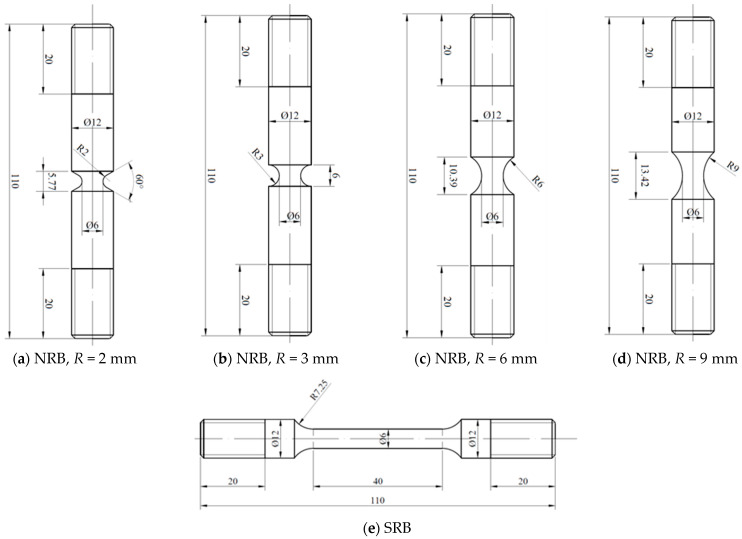
Dimensions of the NRB and SRB tensile specimens.

**Figure 5:**
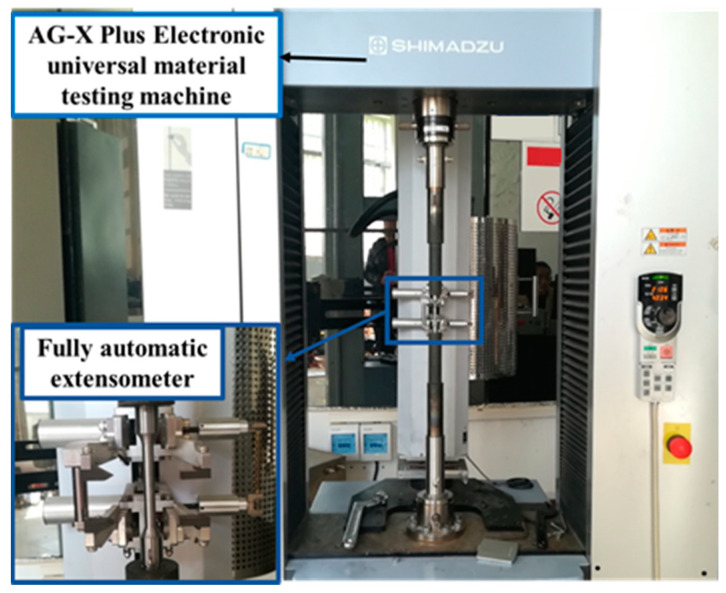
UTM apparatus for the tensile tests.

For the sake of determining the plasticity and fracture behavior of 40CrNiMoA steel in diverse stress conditions, room temperature quasi-static tensile tests were executed using smooth round bar (SRB) and notch round bar (NRB, notch radius *R* = 2 mm, 3 mm, 6 mm and 9 mm) tensile specimens, as shown in Figure 4. For the SRB tensile tests, a 100 kN Shimadzu AG-X plus universal testing machine (UTM, equipped with a ±1% accuracy load cell and a crosshead speed range of 0.001 to 1000 mm/min) together with an extensometer was used, as shown in Figure 5. Tests on the SRB and NRB specimens were conducted in a displacement-controlled manner with a crosshead speed of 1 mm/min applied, giving rise to an average strain rate of 4.2 × 10^−4^ s^−1^ in the gauge section of the SRB specimen.

**Figure 6:**
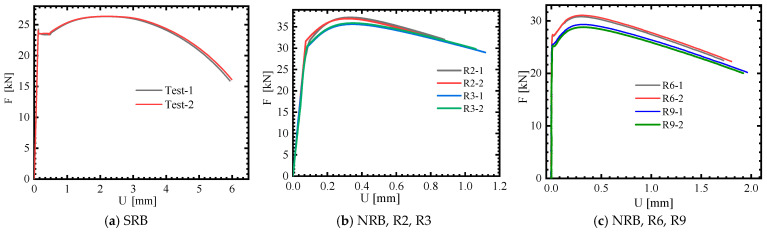
Force–displacement curves of the tensile tests.

**Figure 7:**
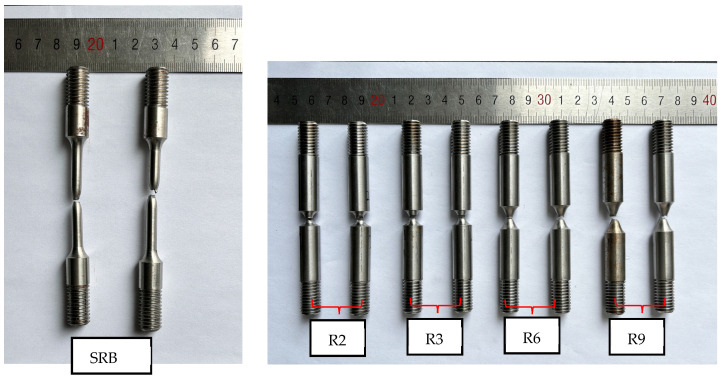
Recovered SRB and NRB specimens tested under quasi-static and room temperature tension.

The final fracture cross-sectional area (*A*_f_) was determined by measuring the diameter at the fracture point (*d*_f_), using the formula *A*_f_ = πd_f_^2^/4. Each testing condition was replicated at least twice, as illustrated in Figure 6. Figure 7 illustrates the recovered round bar specimen, and the fracture morphology clearly indicates that the material experienced ductile fracture. Table 2 lists the 0.2% yield strength (*A*), ultimate strength (*σ*_u_), initial specimen diameter (*d*_0_), fracture cross-section diameter (*d*_f_), area reduction rate (*Ψ*), and fracture strain (ε_f_) for the SRB and NRB specimens. The results from the duplicate experiments demonstrate strong consistency.

It can be seen from Figure 6 a that an obvious Lüders plateau exists in the load–displacement curves of SRB tensile tests; moreover, a less significant Lüders plateau is also observed in the force–displacement curves of the NRB tensile tests, as shown in Figure 6b,c.

### 2.3. SRB Tensile Tests at a Quasi-Static Strain Rate and Elevated Temperature

**Figure 8:**
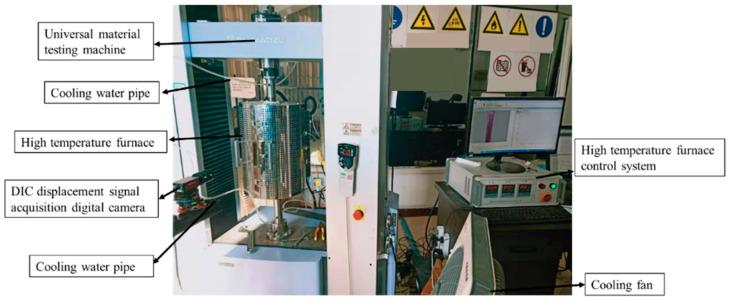
UTM coupled with a heating furnace for tensile tests at elevated temperatures.

To determine the plasticity and fracture behavior of 40CrNiMoA steel at different temperatures, SRB tensile tests were conducted using a UTM coupled with a heating furnace, as shown in Figure 8. Considering the three high temperature values of 300 °C, 500 °C, and 700 °C, the corresponding strain rate was 4.2 × 10^−4^ s^−1^.

**Figure 9:**
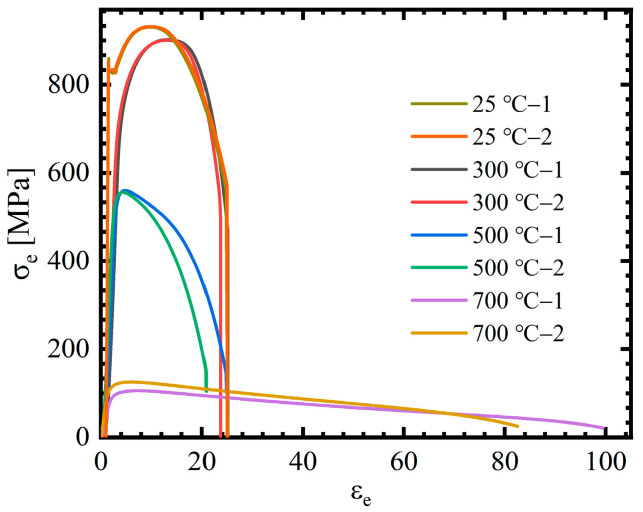
Load–displacement curves of 40CrNiMoA steel at different temperatures.

**Figure 10:**
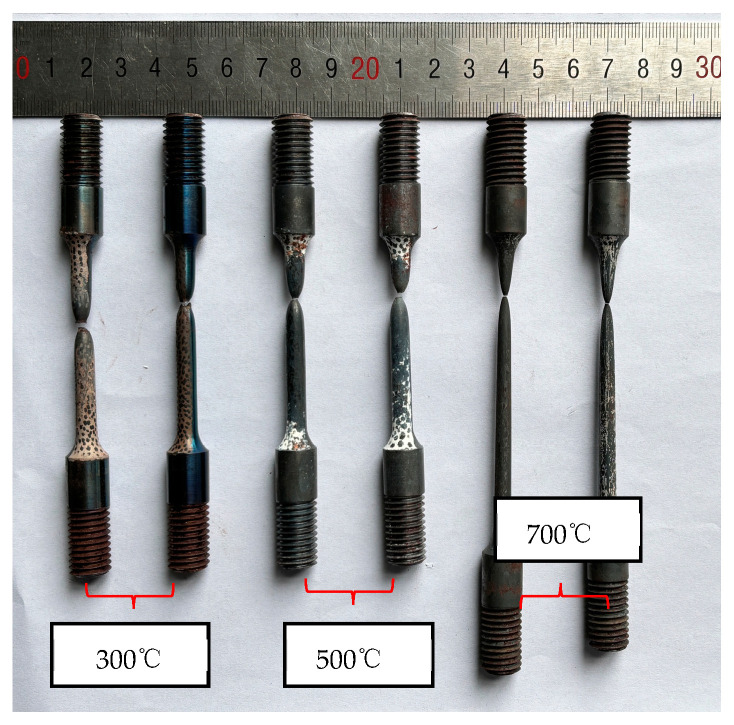
Recovered SRB specimens under tension at a quasi-static strain rate and elevated temperatures.

In elevated temperature tensile tests, the mechanical extensometer is unavailable, so the digital image correlation (DIC) technique is employed to measure specimen displacement. Engineering stress–engineering strain curves of 40CrNiMoA steel at different temperatures are shown in Figure 9. Figure 10 illustrates the recovered SRB specimen, and the fracture morphology clearly indicates that the material experienced ductile fracture. Fracture strain of tensile specimens is calculated by Equation (1) as below:

(1) $$\varepsilon_{f}=\ln\left(A/A_{0}\right)=2\ln\left(d/d_{0}\right)$$

where $A_{0}$ and $d_{0}$ are the cross-sectional area and the diameter of specimens before tensile tests, respectively; while *A* and *d* are the corresponding values after tensile tests.

Yield stress and fracture strain of 40CrNiMoA steel at different temperatures are listed in Table 3. It can be seen that the influence of temperature on the material’s flow stress and fracture strain is minimal at temperatures below 300 °C.; comparatively, at temperatures higher than 500 °C, the yield stress decreases rapidly while fracture shows an opposite tendency.

### 2.4. Dynamic Tests

**Figure 11:**
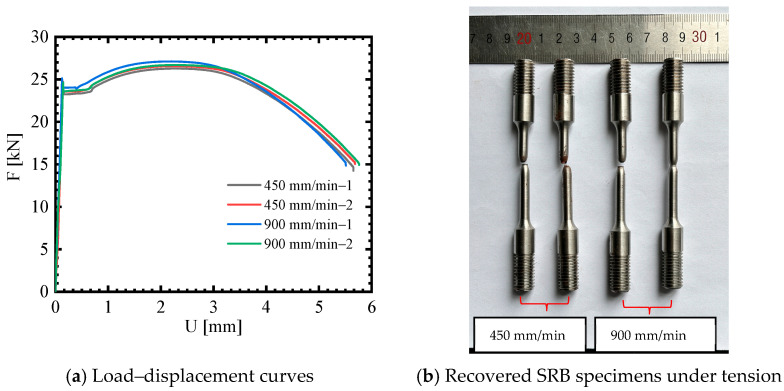
Load–displacement curves and fracture morphologies of the SRB tensile tests at dynamic strain rates.

To obtain the flow stresses and fracture strain of 40CrNiMoA steel at dynamic strain rates, SRB tensile tests at different loading speeds were carried out using a UTM. Two loading speeds of 450 and 1000 mm/min, corresponding to 0.189 s^−1^ and 0.42 s^−1^, respectively, were considered. The test results are shown in Figure 11a. It can be seen that 40CrNiMoA steel does not show obvious strain rate sensitivity within the tested strain rate range. The fracture morphology of post tensile test specimens is shown in Figure 11b, and a cup-shaped fracture pattern, which indicates ductile fracture, was observed. By using Equation (1), the fracture strain for various strain rates was determined and is presented in Table 4.

**Figure 12:**
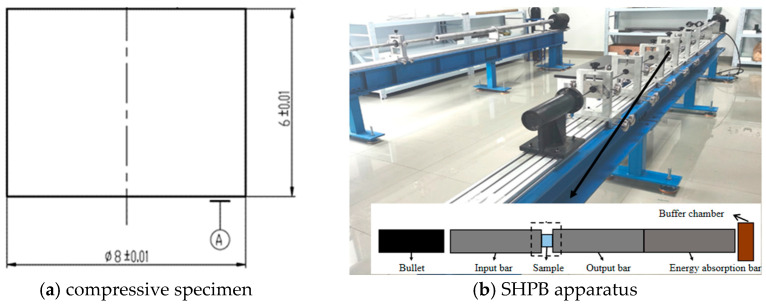
Schematic view of the compressive specimen and SHPB apparatus.

To obtain the flow stresses of 40CrNiMoA steel at even higher strain rates, dynamic compressive tests were carried out with the use of a split Hopkinson pressure bar (SHPB). The compressive specimens are cylindrical in shape, with a diameter of 8 mm and a length of 6 mm, as depicted in Figure 12a. The SHPB consists of an air chamber, bullet, incident rod, transmission rod, energy absorption rod, and buffer chamber, as shown in Figure 12b. All of the bullet (200 mm in length), the incident rod (1400 mm in length), and the transmission rod (1400 mm in length) possess a diameter of 12 mm, and they are all made of Maraging steel C350.

**Figure 13:**
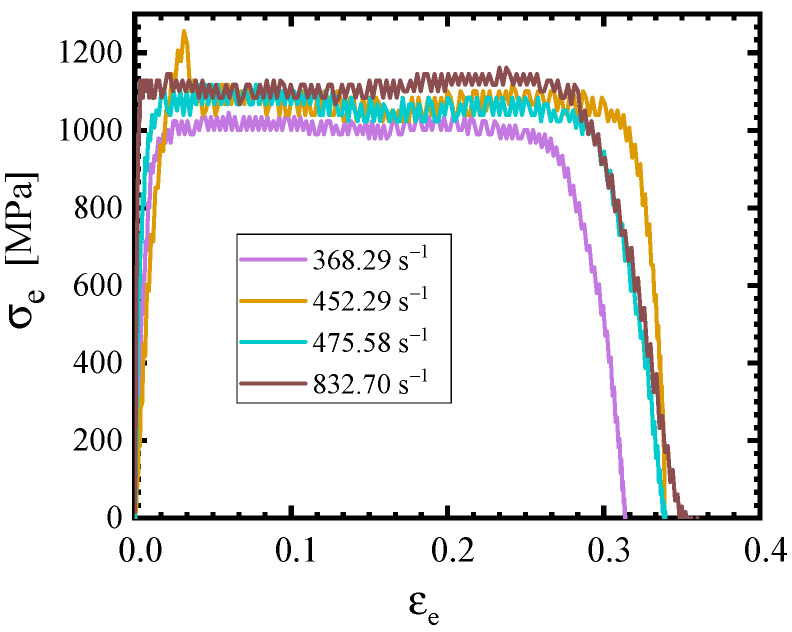
Engineering stress–engineering strain curves of 40CrNiMoA steel at dynamic strain rates.

In the present paper, five strain rates within the region of 368~833 s^−1^ are considered. According to one-dimensional stress wave propagation theory as well as stress equilibrium assumption, engineering stress–engineering strain curves were calculated by Equations (2)–(5), as shown in Figure 13.

(2) $$\dot{\varepsilon }\left(t\right)=-\frac{2C_{0}}{l_{0}}\varepsilon_{r}\left(t\right)$$

(3) $$\varepsilon \left(t\right)=-\frac{2C_{0}}{l_{0}}\int_{0}^{t}\varepsilon_{r}\left(t\right)dt$$

(4) $$\sigma \left(t\right)=\frac{A}{A_{0}}E\varepsilon_{t}\left(t\right)$$

(5) $$\varepsilon_{t}\left(t\right)=\varepsilon_{i}\left(t\right)+\varepsilon_{r}\left(t\right)$$

where *t* is time; $C_{0}$ refers to the velocity of elastic waves in the rod; *l*_0_ stands for the length of the specimen; *A* is the cross-sectional area of the rod; *A*_0_ is the cross-sectional area of the specimen; and $\varepsilon_{i}$, $\varepsilon_{r}$ and $\varepsilon_{t}$ are the strain caused by the incident wave, reflected wave, and transmitted wave, respectively.

Based on the flow stress curves, the yield stresses of 40CrNiMoA steel at different strain rates were calculated and are listed in Table 5, from which an obvious strain rate hardening phenomenon can be observed.

## 3. Material Models and Parameter Calibration

### 3.1. Material Models

In research on impact dynamics, the JC plasticity model has been extensively applied owing to its simplicity. Recently, a modified JC plasticity model with a linear combination of Ludwik’s law [25] and Voce’s law [26] was proposed by Roth et al. [37] to better characterize the plasticity behavior of metallic materials; see Equation (6). Some successful applications can be found in the work of Xiao et al. [23] and Deng et al. [38]. However, it should be noted that neither the MJC plasticity model nor the others take Lüders plateau into consideration. Inspired by this, a plasticity model characterizing Lüders plateau is proposed in the present paper, and this model is referred to as MJC-2. Compared to the MJC plasticity model, this model introduces two model parameters, *e*_0_ and *C*_2_, and a sharp bracket is used in the equivalent. The sharp brackets mean that zero is taken when the value in the brackets is less than zero; this change can maintain the equivalent stress as a constant value when the strain is less than *e*_0_ and thus characterize the hardening behavior of materials with Lüders plateau. The introduction of exponent *C*_2_ exerts an adjustment effect on the rate of ascension of the curve. Subsequent to the supplementation with a yield plateau, the contour of the curve is capable of approximating the real-world situation with enhanced fidelity. Finally, its expression is shown in Equation (7).

(6) $$\sigma_{\mathrm{eq}}=\{\alpha (A+B\varepsilon_{\mathrm{eq}}^{n})+(1-\alpha )[A+Q(1-e^{-C_{1}\varepsilon_{\mathrm{eq}}})]\}(1+C\ln{\dot{\varepsilon }}_{\mathrm{eq}}^{*})(1-pT^{*m})$$

(7) $$\sigma_{\mathrm{eq}}=\left\{\alpha (A+B{\langle \varepsilon_{\mathrm{eq}}-e_{0}\rangle }^{n})+(1-\alpha )[A+Q{\left(1-e^{-C_{1}\varepsilon_{\mathrm{eq}}}\right)}^{C_{2}}]\right\}(1+C\ln{\dot{\varepsilon }}_{\mathrm{eq}}^{*})(1-pT^{{*}^{m}})$$

where *A*, *B*, *n*, $e_{0}$, Q, $C_{1}$, $C_{2}$, C, and m are the model parameters to be calibrated; α acts as a weighting parameter to effectuate the combination of Ludwik’s law and Voce’s law; $\sigma_{\mathrm{eq}}$ is Von Mises stress; $\varepsilon_{\mathrm{eq}}$ represents the equivalent plastic strain; ${\dot{\varepsilon }}_{\mathrm{eq}}^{*}$ denotes the dimensionless strain rate defined as ${\dot{\varepsilon }}_{\mathrm{eq}}^{*}={\dot{\varepsilon }}_{\mathrm{eq}}/{\dot{\varepsilon }}_{0}$, in which ${\dot{\varepsilon }}_{\mathrm{eq}}$ and ${\dot{\varepsilon }}_{0}$ are the current and the reference strain rates, respectively; and T* represents the dimensionless temperature, defined as $T^{*}=\left(T-T_{r}\right)/\left(T_{m}-T_{r}\right)$, in which T, Tr, and Tm are the current, reference, and melting temperatures, respectively.

In the present research, the MJC fracture criterion is employed to characterize the fracture behavior of 40CrNiMoA steel. The criterion has been successfully used in many investigations [23,38], and it is usually shown as:

(8) $$\varepsilon_{f}=\left[D_{1}+D_{2}\exp(D_{3}\eta )\right](1+D_{4}\ln{\dot{\varepsilon }}_{\mathrm{eq}}^{*})(1+D_{5}T^{*D_{6}})$$

where η is stress triaxiality defined as $\eta =\sigma_{m}/\sigma_{\mathrm{eq}}=\left(\sigma_{1}+\sigma_{2}+\sigma_{3}\right)/3\sigma_{\mathrm{eq}}$; $\sigma_{m}$ is hydrostatic pressure; $\sigma_{1}$, $\sigma_{2}$, and $\sigma_{3}$ denote the first, second, and third principal stresses, respectively; and $D_{1}-D_{5}$ are the model parameters.

### 3.2. FE Model Configuration

**Figure 14:**
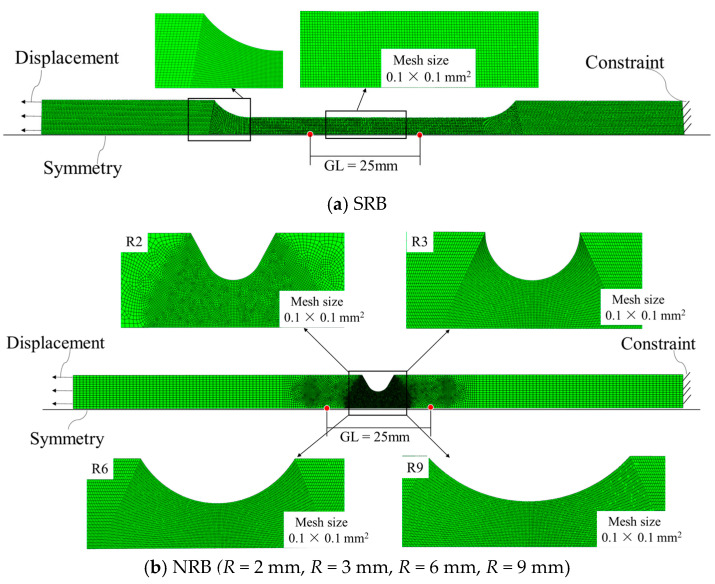
FE models for SRB and NRB tensile tests.

Calibration of the plasticity model and fracture criterion was conducted based on an experimental–numerical approach. FE models for SRB and NRB tensile tests were generated in FE software. Two-dimensional axisymmetric models were established due to their symmetrical features. The models were discretized by the four-node, axisymmetric elements with reduced integration (CAX4R) elements. The FE model was discretized with different mesh densities for different parts of the models. A refined mesh (0.1 mm × 0.1 mm) was used within the gauge section to accurately capture the strain location due to Lüders plateau, while a coarser mesh with smooth mesh transition was used in the other positions. Figure 14 shows the mesh configurations of the FE models and the associated boundary conditions. Nodal displacements were applied at one end of the specimen, while the opposite end was constrained to prevent rigid body motion. The fitted plasticity equation was utilized in the numerical simulation to model the material’s stress flow behavior.

### 3.3. Calibration of the Plasticity Model

The common way to calibrate model parameters is to decouple the model and then calibrate each multiplier separately [23]. This method needs limited experimental data and saves a lot of costs; thus, it is used in the present paper.

As is evident from Equation (6), the plasticity model we have employed is of the Von Mises plasticity type. Since it does not take the stress state into account, in this section, it is initially calibrated using the uniaxial tensile test data of the SRB. The quasi-static uniaxial tensile test of the SRB was conducted at room temperature. Under such circumstances, the plasticity model is expressed as:

(9) $$\sigma_{\mathrm{eq}}=\alpha (A+B\varepsilon_{\mathrm{eq}}^{n})+(1-\alpha )[A+Q(1-e^{-C_{1}\varepsilon_{\mathrm{eq}}})]$$

(10) $$\sigma_{\mathrm{eq}}=\left\{\alpha (A+B{\langle \varepsilon_{\mathrm{eq}}-e_{0}\rangle }^{n})+(1-\alpha )[A+Q{\left(1-e^{-C_{1}\varepsilon_{\mathrm{eq}}}\right)}^{C_{2}}]\right\}$$

where yield strength *A* is taken from the stress at Lüders plateau, and the other parameters (*B*, $e_{0}$, *n*, *Q*, $C_{1}$, and $C_{2}$) can be determined through fitting the true stress–strain curve prior to the initiation of necking [39]. α is optimized by using the numerical iterative calculation of SRB tension, and the optimized value that makes the numerical stress–strain curve agree with that in the experiment is the α we need. 

**Figure 15:**
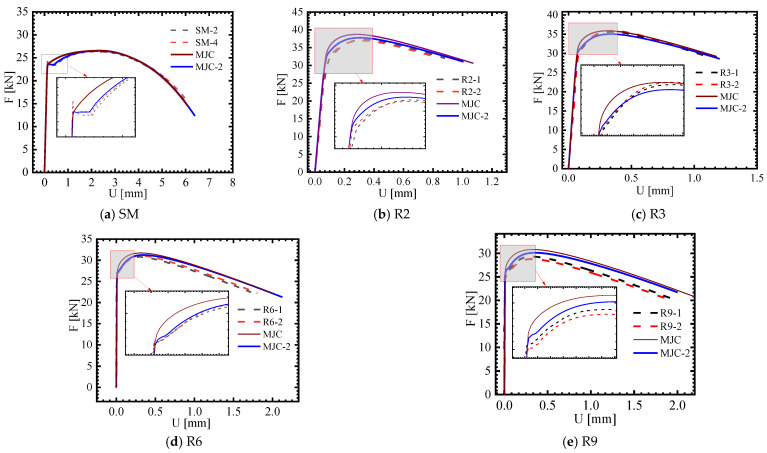
Numerical simulation results for SRB and NRB tensile tests.

Figure 15 shows the numerical simulation results of the SRB and NRB tensile tests. It can be seen that the MJC-2 plasticity model better characterizes the flow stress of 40CrNiMoA than the MJC plasticity model does, and it also reproduces Lüders plateau accurately. By comparing the simulated load–displacement curves of the NRB with the test data, it can be seen that the numerical simulation can predict yield inflection points or a short Lüders plateau, which indicates that the MJC-2 plasticity model can predict the flow stress behavior of the material under different stress states more accurately.

**Figure 16:**
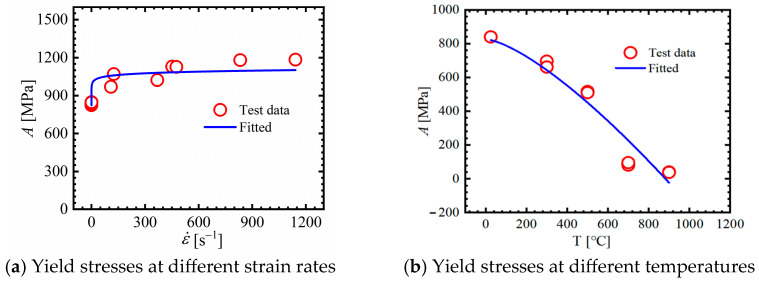
Yield stresses at varying strain rates and temperatures as well as the fitting result (approximation equation: $\sigma_{\mathrm{eq}}=(1+0.022\ln{\dot{\varepsilon }}_{\mathrm{eq}}^{*})$ for (**a**) and $\sigma_{\mathrm{eq}}=(1+2.064*T^{*1.31})$ for (**b**)). Note: In the figure (**a**), the first two values are obtained from tensile tests, whereas the other is obtained from compressive tests.

Dynamic compression tests were used to identify the strain rate hardening coefficient in the plasticity model. By fitting yield stresses at different strain rates, *C* = 0.022 was obtained, as shown in Figure 16a.

Similarly, the yield stress in the SRB tensile tests under different temperatures was employed to identify the temperature softening coefficient. And the thermal softening effect constants *p* and *m* can be achieved through fitting the above data on the basis of the least squares method. The temperature term parameters *p* and *m* were found to be 2.064 and 1.31, respectively, and the fitting result is illustrated in Figure 16b.

### 3.4. Calibration of the Fracture Criterion

When calibrating the fracture criterion, it is crucial to determine the fracture strain in a variety of stress states, strain rates, and temperatures. However, the fracture strain corresponding to various stress states cannot be obtained simply by measuring the cross-sectional area of the NRBs; consequently, a combined strategy of experimental and numerical analysis was employed. Figure 14 shows the FE models for the SRB and NRBs, and the calibrated plasticity model mentioned above is utilized to conduct numerical simulations. Figure 15 presents the numerically simulated load–displacement curves of the SRB and NRBs. It can be observed that the results of the FE simulations are in close agreement with the relevant experimental results. In this circumstance, it is reasonable to determine the fracture strain under various stress states from the FE simulation results, relying on the consistency assumption. It is evident from Figure 15 that the flow stress predictions of 40CrNiMoA using the MJC and MJC-2 plasticity models differ, which inevitably leads to different fracture strain predictions. Consequently, the MJC fracture criterion is calibrated in two ways:

(1) Using fracture data obtained according to numerical simulations by the MJC plasticity model.
(2) Using fracture data obtained according to numerical simulations by the MJC-2 plasticity model.

In the load–displacement curves, the moment of fracture initiation is typically identified by a sudden drop in load; thus, the equivalent plastic strain (PEEQ) and stress triaxiality of the central element in numerical simulations at the moment of fracture initiation were extracted as fracture data. Furthermore, during tensile tests, the necking phenomenon leads to a change in stress triaxiality within the central element of the tensile specimen as strain accumulates [14]. Therefore, the average stress triaxiality is adopted:

(11) $${\bar{\eta }}_{av}=\frac{1}{\varepsilon_{f}}\int_{0}^{\varepsilon_{f}}\eta (\varepsilon_{\mathrm{eq}})d\varepsilon_{\mathrm{eq}}$$

**Figure 17:**
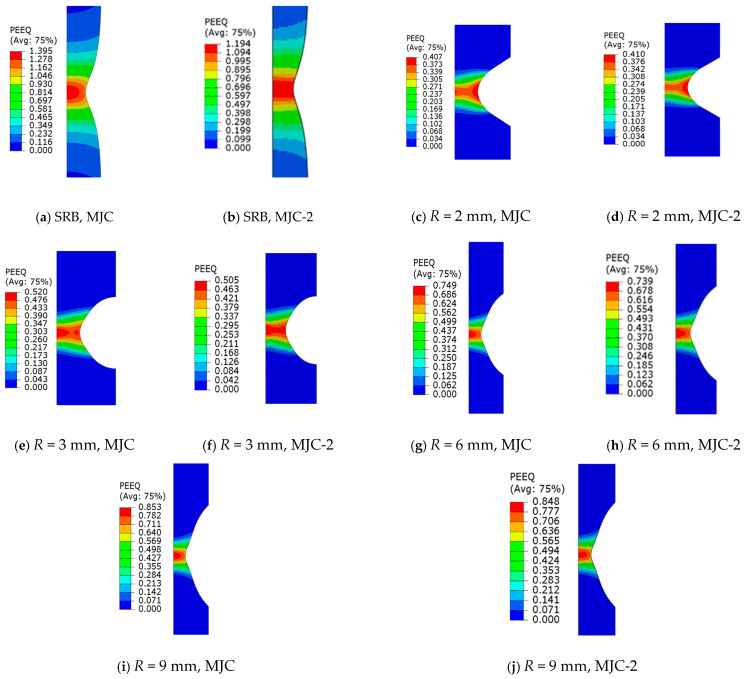
Distribution of the equivalent plastic strain of SRB and NRB specimens at the fracture initiation point.

**Figure 18:**
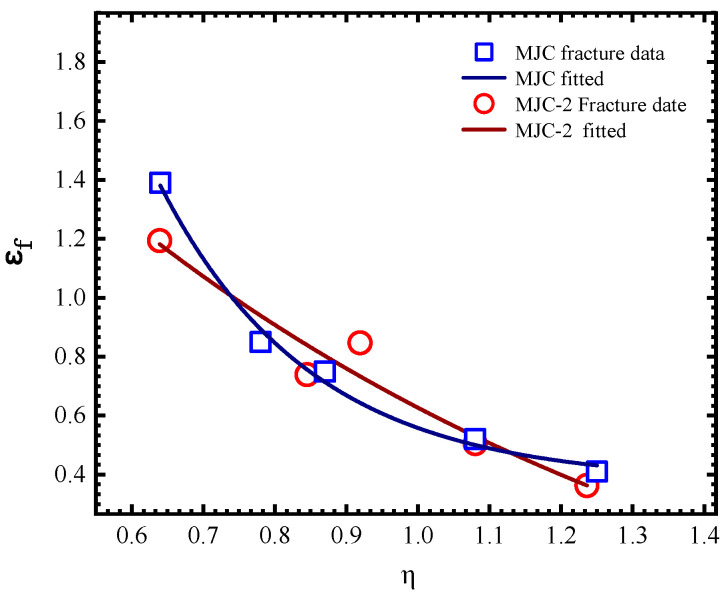
Fracture strain prediction by the MJC and MJC-2 plasticity criteria (approximation equation: $\varepsilon_{f}=0.403+75.6*\exp(-6.397*\eta )$ for MJC and $\varepsilon_{f}=-0.567+3.437*\exp(-1.057*\eta )$ for MJC-2).

Figure 17 shows the PEEQ cloud diagrams of the SRB and NRBs at the moment of fracture initiation. It is evident that the maximum PEEQ appears at the central element; therefore, the PEEQ value of the central element was selected and taken as the fracture strain. Table 6 compiles all fracture data identified through the combined experimental–numerical approach mentioned above. Utilizing these fracture data and the quasi-static term of the MJC fracture criterion outlined in Equation (8), we derive two distinct sets of fracture criterion parameters using the least squares method, which we refer to as the MJC fracture criterion and the MJC-2 fracture criterion. The curve representing the fitting results is displayed in Figure 18. It can be seen that for low and high stress triaxialities, the MJC criterion predicts a higher fracture strain compared to the MJC-2 criterion, while for the other stress triaxialities, an opposite tendency is observed. Different fracture strain predictions by the two criteria caused different numerical simulations of ballistic impact behavior, and this point will be discussed in detail later.

**Figure 19:**
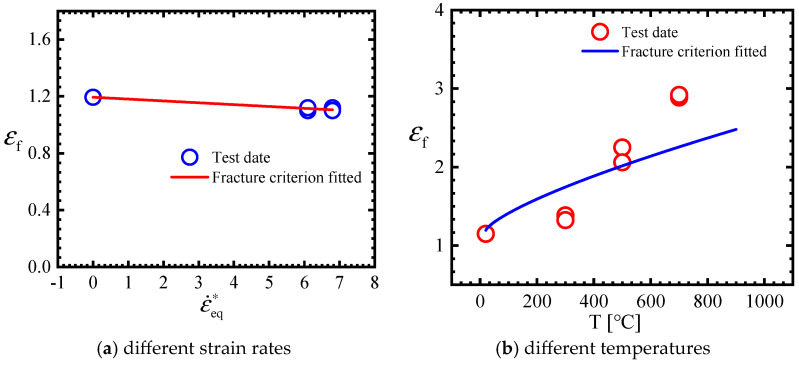
The fitting results at different strain rates and different temperatures (approximation equation: $\varepsilon_{f}=(1-0.04\ln{\dot{\varepsilon }}_{\mathrm{eq}}^{*})$ for (**a**) and $\varepsilon_{f}=(1+1.6*T^{*0.74})$ for (**b**)).

Similarly, the strain rate sensitivity and temperature sensitivity parameters of the MJC and MJC-2 fracture criteria were derived by fitting the fracture strain data across various strain rates and temperatures. Figure 19 compares the experimental data with the fracture criterion predictions. It should be noted that due to a lack of available apparatus, fracture strain data at high strain rates are missing. Therefore, the strain rate-sensitive parameter obtained in the present study is the maximum available approximation. All material parameters are shown in Table 7 and Table 8.

## 4. Taylor Impact Tests and the Corresponding Numerical Simulations

**Figure 20:**
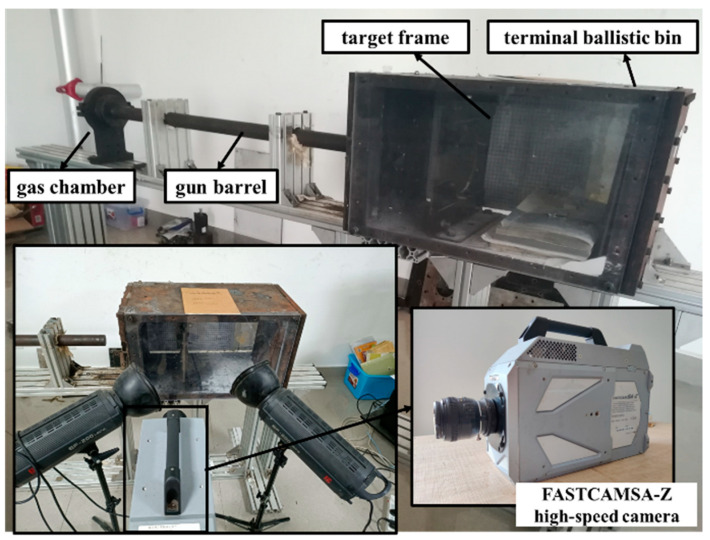
One-stage light gas gun device.

It is known that large strain, high strain rate, and elevated temperature occur during Taylor impact tests, and this technique can be used as an effective method to verify established plasticity models and fracture criteria [40]. The specimen for the Taylor impact test is a cylinder measuring 5.95 mm in diameter and 29.75 mm in length. A one-stage gas gun at Nanyang Institute of Technology was utilized for the Taylor impact tests. The setup includes a pressure chamber, a launch tube, and an impact chamber, as depicted in Figure 20. The projectile’s initial velocity is regulated by varying the compressed gas pressure. Additionally, the Photron FASTCAM SA-Z high-speed camera captures the impact process at 60,000 fps, with the projectile’s initial velocity (*V*_0_) calculated by the time interval and distance between consecutive frames.

**Figure 21:**
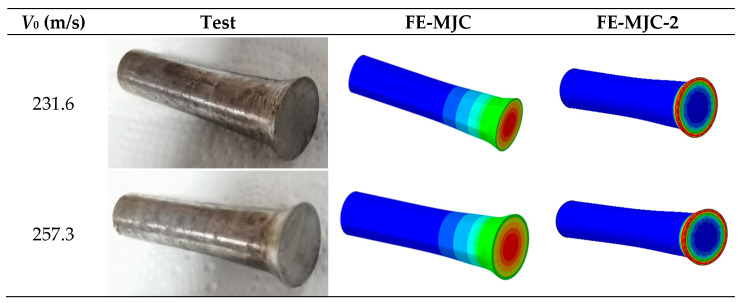
Final shapes of specimens at different impact velocities.

Figure 21 show the final shapes of specimens at different impact velocities. It can be seen that for low impact velocities, a mushroom shape forms at the head of the specimens; for high impact velocities, cracks are observed at the head of the specimens. With a further increase in impact velocity, a petal-shaped fracture pattern forms, indicating a circumferential stress stretching tear rather than shear fracture, and a similar phenomenon was observed in the study of Wei et al. [40]. For specimens without fracture, the residual specimen diameter (*d_f_*) and head length (*l_f_*) were measured and are shown in Table 9.

**Figure 22:**
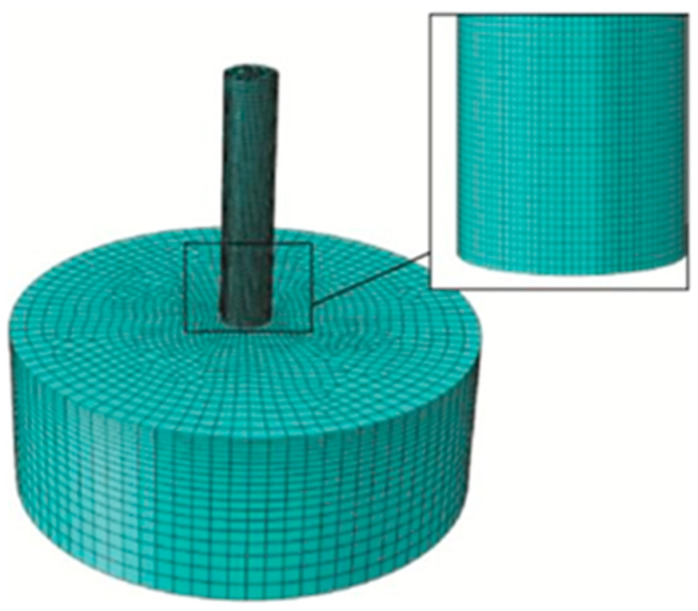
FE model of Taylor impact tests.

To verify the previously established plasticity model and fracture criterion, numerical simulations of Taylor impact tests were conducted using FE software. In FE analysis, the stress, strain, and deformation were determined at the intersection of the mesh. Therefore, to ensure analysis accuracy, selecting the appropriate element type and size is crucial. Solid, three-dimensional, eight-node elements with linear displacement approximation, three translational degrees of freedom, and reduced integration with hourglass control (C3D8R) are suitable for both linear and nonlinear models. These elements can manage large deformations, accommodate plasticity, and accurately incorporate contact properties. Therefore, in this study, FE analyses were carried out using the C3D8R element type. The plasticity and fracture behavior of Taylor rods are described by the established plasticity and fracture criterion, while the targets are regarded as rigid. The “hard” contact algorithm was used to simulate contact behavior between the Taylor rods and the targets, and the friction phenomenon was ignored, as shown in Figure 22.

To verify mesh sensitivity, different mesh sizes (0.5 × 0.5 × 0.5 mm^3^, 0.2 × 0.2 × 0.2 mm^3^, 0.1 × 0.1 × 0.1 mm^3^, and 0.05 × 0.05 × 0.05 mm^3^) were used for the Taylor rods, while the mesh size of the target remained unchanged as 0.5 × 0.5 × 0.5 mm^3^. The numerical simulation results are compared with the experimental results, as shown in Figure 21 and Table 9.

**Figure 23:**
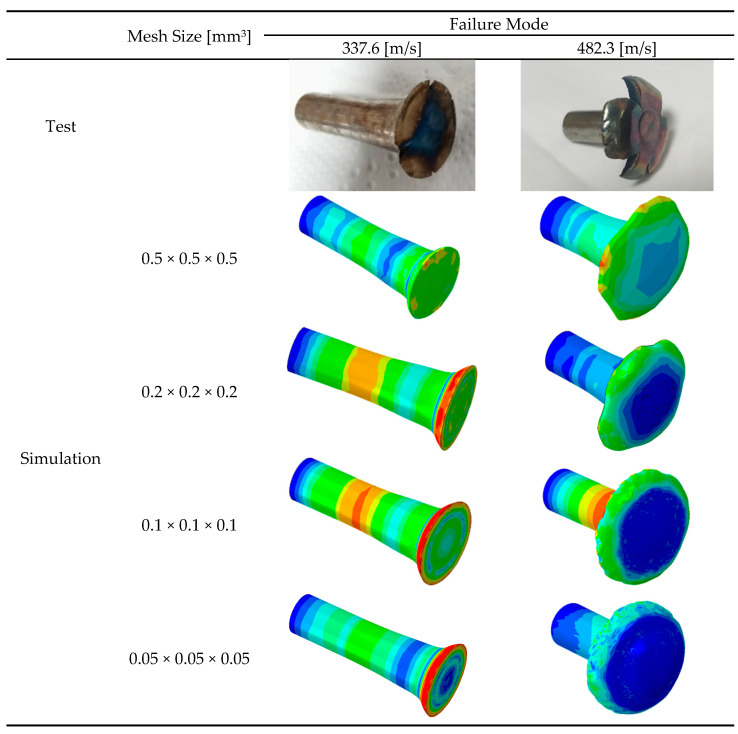
Taylor rod impact test and simulation results at different mesh sizes.

**Figure 24:**
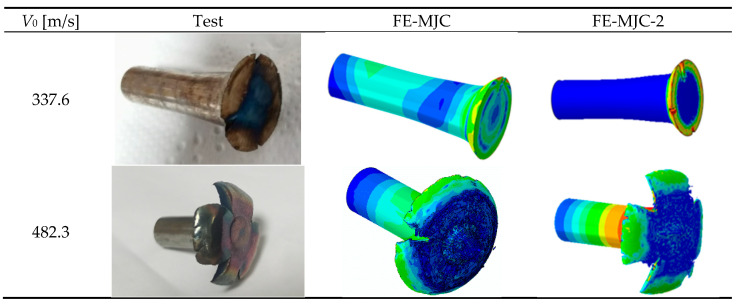
The simulation results of Taylor rods when *D*_4_ = −0.04.

The experimentally obtained shapes of the Taylor rods were added to the numerical ones at 231.6 m/s and 257.3 m/s, as shown in Figure 21. It was found that the numerical shapes of Taylor rods using the MJC-2 plasticity model were closer to the experimental results, with errors smaller than 10%, as shown in Table 9. However, neither of the two fracture criteria predict the fracture behavior of Taylor rods at higher impact velocities. One possibility is that numerical simulations of high-speed impact events are highly sensitive to mesh size, as reported by Børvik et al. [41]. Therefore, different mesh sizes were used for Taylor rods, and the numerical results were added to Figure 23. Evidently, the fracture behavior of Taylor rods cannot be predicted at any of the mesh sizes. This seems to be in contradiction with our previous study [42], in which the ballistic impact behavior of high-strength steel targets against blunt projectiles was accurately predicted with finer mesh sizes. Another possibility is that strain rate sensitivity parameter *D*_4_ in the fracture criterion is inaccurate as the value is calibrated according to experiments over a pretty limited strain rate range. Therefore, the value of $D_{4}$ was recalibrated by the fitting numerical fracture patterns to the experimental ones. It was found that $D_{4}$ = −0.04 can successfully predict the experimentally obtained four-petal cracking patterns, as shown in Figure 24. This also successfully verifies the validity of the MJC-2 fracture criterion. However, the numerical fracture patterns using the MJC fracture criterion are still different from the experimental ones regardless of $D_{4}$ = −0.04. Therefore, we can draw the conclusion that Lüders plateau is of great significance in the fracture analysis of metals under high-speed impact.

## 5. Ballistic Impact Tests and Numerical Simulations

### 5.1. Experimental Work

To investigate the influence of incorporating Lüders plateau into the plasticity model on predicting the ballistic impact behavior of 40CrNiMoA, we conducted ballistic impact tests. The device used in the ballistic impact tests is the same as that used in the Taylor impact tests, as shown in Figure 20. To avoid plastic deformation during ballistic testing, hardened 9CrSi steel projectiles with a hardness of 52HRC were used, and the experimental projectile had a flat-headed cylindrical shape with a nominal diameter, length, and mass of 5.96 mm, 29.82 mm, and 6.40 g, respectively. The target was a circular plate with a thickness of 4 mm and a diameter of 42 mm and its machining used the same batch of materials as in mechanical testing.

**Figure 25:**
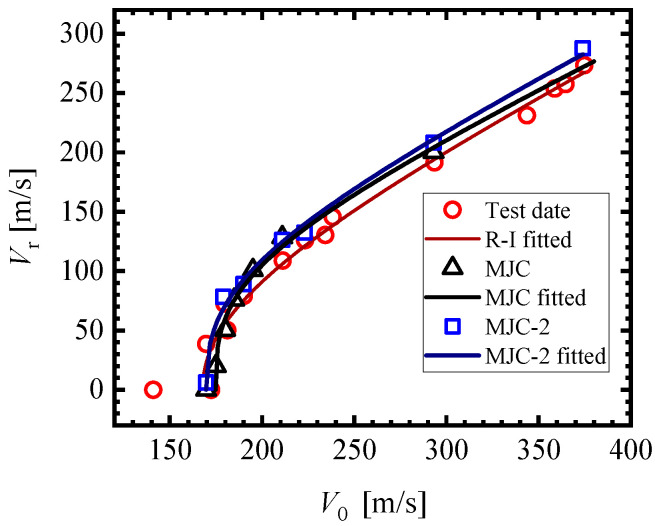
Ballistic response of the penetration test and numerical simulation.

Fifteen effective ballistic impact tests were carried out at speeds ranging from 114.7 m/s to 374.8 m/s. Figure 25 shows the initial and residual velocities of the projectiles. By using these data, the BLVs and ballistic curves were calculated using the equation proposed by Recht and Ipson [43], and the Recht–Ipson(R-I) equation is as follows:

(12) $$Vr=a{\left(V_{0}^{p}-{V_{\mathrm{bl}}}^{p}\right)}^{1/p}$$

where *a* and *p* are model constants that define the curvilinear path; $V_{0}$ and $V_{r}$ denote the initial and residual velocities of the projectile, respectively; and $V_{bl}$ represents the ballistic limit velocity. By using the least squares method to run the best fit of the initial residual velocities, we obtained the BLV: *V*_Test_ = 167.2 m/s.

**Figure 26:**
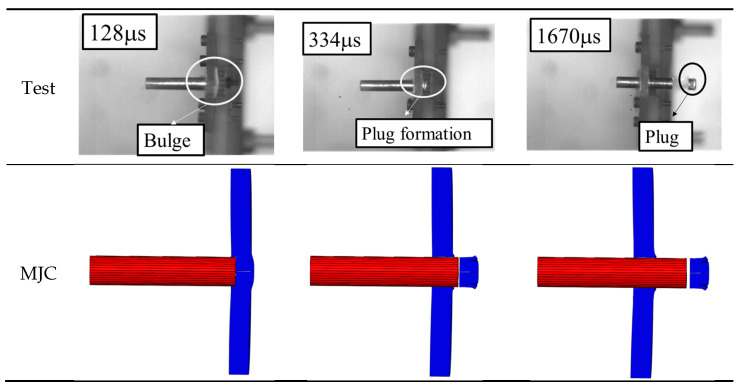

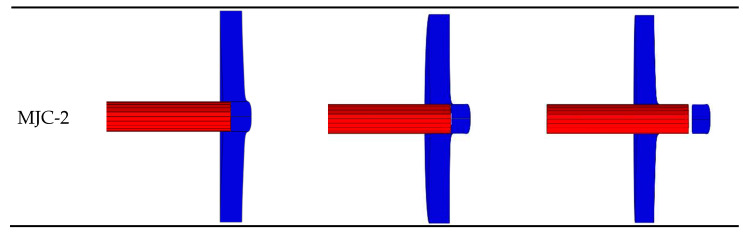
The failure process of the target at an impact velocity of 190 m/s.

**Figure 27:**
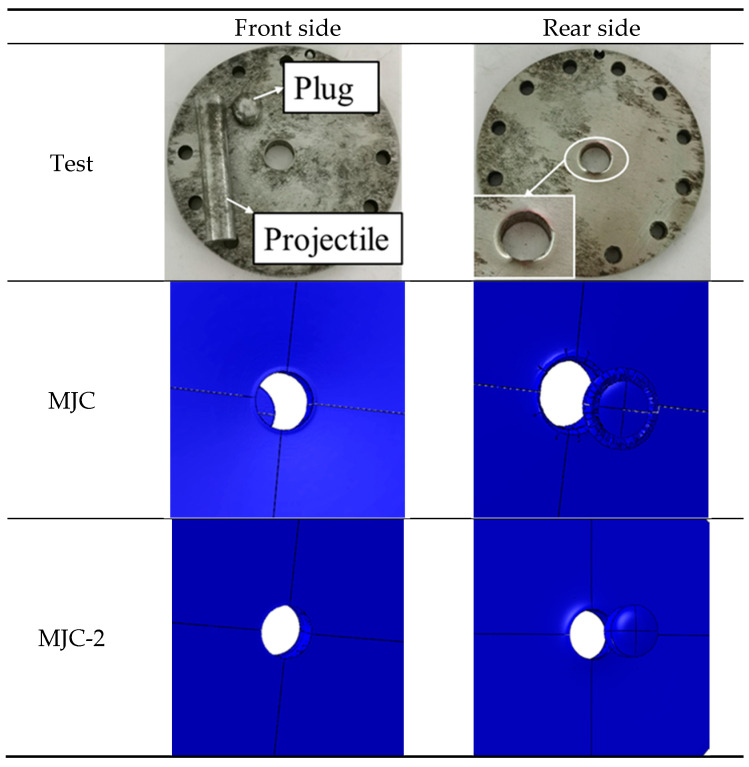
The failure mode of the target at an impact velocity of 190 m/s.

Figure 26 shows the ballistic impact process captured by a high-speed camera. It can be seen that the projectile has a good flying attitude without any obvious deviation. Under the impact of the flat-nosed projectile, the target first produces a bulge on the back and is then penetrated by the projectile and produces a complete cylindrical plug without any fragments. By observing the recovered target, the shear plugging failure mode was found, as shown in Figure 27, which indicated that severe plastic deformation is restricted to a narrow region, leaving the rest of the plate nearly undeformed.

### 5.2. Numerical Simulations

**Figure 28:**
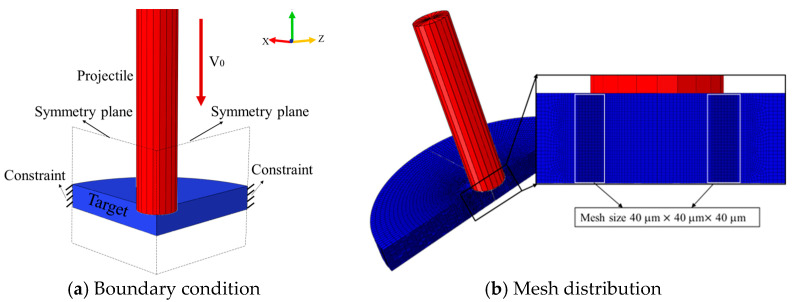
Simulation model configuration.

A quarter 3D simulation model with a 3D8RX element corresponding to the ballistic impact test was established using FE software; the target is defined as deformable bodies, while the projectile is regarded as rigid. For the target, the nodes on the outer circle are completely constrained, while the projectile is free to move only in the axial direction, and the projectile is the given initial velocity consistent with the experiment to run numerical simulations. A transition mesh was adopted in the FE model, and in the target, the finest mesh size with an average length of 40 μm^3^ was used in the region where the shear failure may occur, while the mesh size further away from this region increases sequentially; in the projectile, the mesh size is about 0.5 × 0.5 × 0.5 mm^3^, as shown in Figure 28. This meshing method can not only ensure the fineness of the grid but also improve the efficiency of the analysis. A similar element strategy has verified the accuracy of numerical simulation for other high-strength steels; see ref. [42]. For the interaction between the projectile and the target, “Hard” contact was adopted. The friction on the contact surfaces was neglected, and an initial separation distance of 0.1 mm was specified between the projectile and the target.

In numerical simulations, the MJC-2 plasticity model was utilized to describe the plasticity of the material, while the MJC-2 fracture criterion was used to describe its fracture behavior. In addition, to investigate the influence of Lüders plateau on predicting the ballistic impact behavior of a 40CrNiMoA steel plate, a combination of the MJC plasticity model and MJC fracture criterion was also employed. Moreover, this combination will be used as a comparison option to further validate our results.

Figure 25 illustrates the initial and residual velocities of the projectile from two simulation series. By using the R-I model (Equation (12)) to run the best fit of the initial residual velocities, we obtained the BLV: *V*_MJC-2_ = 169.7 m/s and *V*_MJC_ = 174.8 m/s. Obviously, the BLV simulated using the MJC-2 model is consistent with the test, whereas the BLV is overestimated using the MJC model.

The failure mechanisms predicted by the MJC fracture criterion and MJC-2 fracture criterion were compared with those in the experiment, where a representative impact velocity that is slightly higher than the BLV is considered. Figure 26 illustrates the failure process of the target, while Figure 27 shows the corresponding failure mode. Subsequently, the following conclusions can be drawn: when the projectile impacts the target, the interaction between the projectile and the plate causes deformation of the target during a short period of time and then a bulge begins to rise on the back of the target. Subsequently, an intact cylindrical plug is flushed out of the target, leaving a clean hole in the target without any cracks found around the hole. This impact process is accurately predicted by the MJC-2 model. However, in MJC-based simulation, cracking of the target occurs before the projectile penetrates it, and with further movement of the projectile, the petal-shaped plug is ejected from the target; moreover, small petal-like fragments are detected around the hole in the target. This is exactly contrary to the experimental results. From this, it can be seen that the MJC-2 model exhibits more accurate predictions than the MJC model.

## 6. Discussion

Figure 15 clearly shows that the MJC-2 plasticity model correctly describes the deformation behavior of 40CrNiMoA under different stress triaxialities due to the existence of Lüders plateau. Meanwhile, we can draw a conclusion from Figure 17 that Lüders plateau significantly influences plasticity evolution in FE analysis. Hence, it is noteworthy that the fracture strain under different stress triaxialities depends on the results of FE analysis incorporating the plasticity relation only. This prompted us to use two different fracture data to calibrate the MJC fracture criterion, so as to further explore the influence of Lüders plateau on the numerical simulation of the material under high-speed impact. Figure 26 and Figure 28 show the numerical simulation results using the MJC model and the MJC-2 model. By making a comparison between the numerical results considering BLV and fracture modes and those experimentally obtained, we can conclude that the FE simulations using the MJC-2 fracture criterion considering Lüders plateau give almost the same BLV and fracture modes as that in the experiment. However, the BLV is overestimated by using the MJC criterion, and the predicted target fracture mode is also completely different from the experiment.

**Figure 29:**
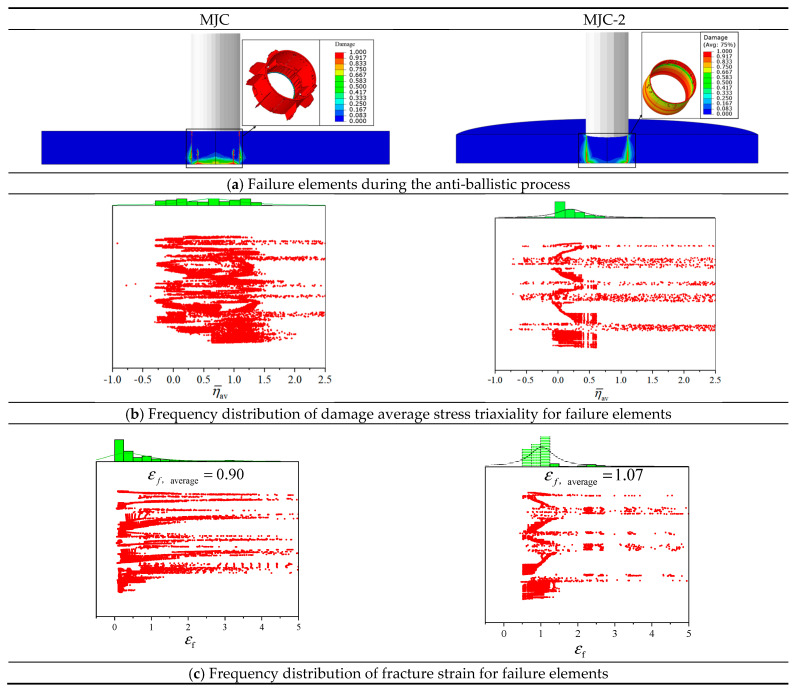
Failure element analysis illustration.

To analyze the influence of Lüders plateau on the numerical simulation of the ballistic impact behavior of 40CrNiMoA, the stress triaxiality and fracture strain of the failure element during numerical simulation were analyzed. The numerical simulation at 190 m/s impact velocity was selected to extract the average stress triaxiality of the failure element. Referring to the work of Xiao et al. [44], the average stress triaxiality is defined by

(13) $${\bar{\eta }}_{D}=\frac{1}{D_{f}}\int_{0}^{D_{f}}\eta (D)\;dD$$

where d*D* is the increment of damage index *D* in one calculation increment and $D_{f}$ represents the element’s damage indicator at the calculation’s conclusion. This characterization strategy more effectively captures the main stress state in the process of damage accumulation. Figure 29b shows the damage stress triaxiality frequency distribution of the failure element during the ballistic process. According to the frequency distribution diagram, the damage average stress triaxiality using the MJC model is mainly in the range $0<{\bar{\eta }}_{D}<1.49$, while most of the failure elements lie among damage average stress triaxiality using the MJC-2 model. In terms of MJC simulation results, the failure elements with stress triaxiality greater than 1 account for almost half of the total failure elements, which means that the target failure mode predicted by the MJC model includes not only shear failure but also tensile failure, which is contrary to the experimental shear plugging failure mode. However, for the MJC-2 numerical simulation results, the stress triaxiality of almost all of the failure elements is close to 0, which is exactly the shear stress state.

Figure 29c shows the fracture strain frequency distribution of failure elements. It can be seen that whether the MJC or MJC-2 model is used for numerical simulation, the fracture strain of all of the failure elements is in the range of 0.1~1.4, but the average fracture strain makes a difference. The average fracture strain of the MJC model is 0.90, which is lower than that of the MJC-2 model (1.07), so the prediction results of the MJC-2 model exhibited better ductility, which is consistent with the observed experimental results. Moreover, in the results of the MJC numerical simulation, the number of failure elements was much higher than that of the MJC-2 model, and MJC predicted a failure of 22,881 elements, while MJC-2 predicted a failure of 13,697 elements, where the number of failure elements predicted by MJC is almost twice as many as that of MJC-2. For the target resistance to blunt-nosed projectiles, the failure mode is shear plugging, which results in severe plastic deformation in a relatively narrow area, with a proportionally limited amount of material deformation or failure. While the failure elements predicted by the MJC model extend to the plug, causing the number of failure elements to double, this is obviously inconsistent with the experimental results.

In summary, through the above analysis and comparison of the numerical results of the two series, the application of Lüders plateau in numerical simulation has been proven to be effective in capturing the crack propagation of ductile materials under high-speed impact and can predict the appropriate conservative BLV, which improves the accuracy and reliability of numerical simulations.

## 7. Conclusions

In this study, comprehensive mechanical tests were carried out on 40CrNiMoA steel that exhibited the Lüders plateau phenomenon. In using the MJC-2 model to simulate Lüders behavior, we found that Lüders plateau has a significant effect on the strain field of the SRB and NRB and the global structural response of the target. The following conclusions can be drawn:

- The traditional MJC model cannot reproduce Lüders plateau in tensile stress–strain curves, and the MJC-2 plasticity model incorporating Lüders plateau was established and the model was able to describe Lüders plateau accurately.
- The failure strain field of SRB and NRB tensile tests are sensitive to Lüders plateau; therefore, the model should be carefully selected for calibration to perform the appropriate conservative crack estimation.
- The MJC-2 model provides a more accurate prediction of the target failure mode and BLV, primarilydue to the existence of Lüders plateau, which accurately determines the strain at the crack tip.

The development of the MJC-2 plastic model significantly enhances the predictive accuracy of impact-induced damage evolution in critical structural components and facilitates the optimization of impact-resistant designs. This advanced modeling approach exhibits substantial potential for engineering applications across multiple domains, particularly in aerospace engineering for spacecraft shielding optimization, the automotive industry for crashworthiness assessment, and the defense sector for ballistic protection system design, thereby contributing to improved structural integrity and safety performance in these technologically demanding fields. Future work should focus on optimizing the MJC-2 model, expanding its applicability, and developing advanced tools for Lüders plateau analysis under various loading conditions.

## Figures and Tables

**Table 1:** Chemical composition of 40CrNiMoA steel (wt.%).

Composition	C	Si	Mn	P	S	Ni	Cr	Mo
Percentage content (min)	0.37	0.17	0.5	—	—	0.25	0.6	0.15
Percentage content (max)	0.44	0.37	0.8	0.025	0.025	0.65	0.9	0.25

**Table 2:** Main result for the SRB tensile test.

Specimen	No.	*E* [GPa]	*A* [MPa]	$\sigma_{u}$ [MPa]	*d*_0_ [mm]	*d*_f_ [mm]	*Ψ* [%]	$\varepsilon_{f}$
SRB	1	184.30	825.30	959.80	6.00	4.46	46.6	1.16
	2	176.50	816.58	970.60	6.01	4.40	45.1	1.14
NRB, *R* = 2 mm	1	-	-	-	6.01	5.36	20.5	0.40
	2	-	-	-	5.99	5.40	18.9	0.42
NRB, *R* = 3 mm	1	-	-	-	6.00	5.40	20.4	0.53
	2	-	-	-	6.01	5.35	19.5	0.51
NRB, *R* = 6 mm	1	-	-	-	6.01	5.22	24.3	0.76
	2	-	-	-	5.99	5.18	25.6	0.74
NRB, *R* = 9 mm	1	-	-	-	6.00	5.15	25.1	0.85
	2	-	-	-	5.99	5.19	26.2	0.85

**Table 3:** Results of high-temperature tensile tests conducted on the SRB.

*T* [°C]	*No.*	*A* [MPa]	$\sigma_{u}$ [MPa]	*d*_0_ [mm]	*d*_f_ [mm]	*Ψ* [%]	$\varepsilon_{f}$
25	1	822.3	965.6	6.01	4.46	46.6	1.16
	2	820.5	960.4	6.00	4.40	45.1	1.14
300	1	690.5	952.3	5.99	4.25	49.7	1.35
	2	696.5	950.5	6.00	4.36	48.5	1.39
500	1	517.8	568.6	6.01	3.39	75	2.25
	2	519.8	565.7	6.00	3.56	68	2.30
700	1	81.4	92.7	5.99	2.02	88.5	2.82
	2	88.5	99.5	6.01	1.94	89.5	2.90

**Table 4:** Under tensile conditions, the fracture strain of 40CrNiMoA steel at different strain rates.

$\dot{\varepsilon }$ [s^−1^]	4.2 × 10^−4^	0.189	0.189	0.420	0.420
$\varepsilon_{f}$	1.19	1.10	1.12	1.12	1.1

**Table 5:** Yield stresses of 40CrNiMoA steel at different strain rates.

$\dot{\varepsilon }$ [s^−1^]	0.0004	0.19	0.42	368.29	452.29	475.58	832.70
*A* [MPa]	841	833	849	1023	1129	1126	1180

**Table 6:** Fracture strain and stress states for different NRB tensile specimens.

**Test Configuration**	***u*_f_ [mm]**	${\bar{\eta }}_{\mathrm{av}}$		$\varepsilon_{f}$	
		**MJC**	**MJC-2**	**MJC**	**MJC-2**
R = 2	0.82	1.25	1.24	0.41	0.41
R = 3	1.12	1.08	1.08	0.52	0.51
R = 6	1.72	0.87	0.85	0.75	0.74
R = 9	1.94	0.78	0.92	0.85	0.85
R = ∞	5.99	0.64	0.64	1.39	1.19

**Table 7:** Parameters of the MJC model.

*E* [GPa]	*v*	*ρ* [kg/m^3^]	*A* [MPa]	*B* [MPa]	*n*	*A*	*Q* [MPa]	$C_{1}$	C	${\dot{\varepsilon }}_{0}$ [s^−1^]
80.4	0.3	7800	820.94	1107.19	0.28	0.42	211.34	25.24	0.022	4.2 × 10^−4^
*p*	*m*	$T_{0}$ [K]	$T_{m}$ (K)	$C_{p}$ [J/kg K]	$D_{1}$	$D_{2}$	$D_{3}$	$D_{4}$	$D_{5}$	$D_{6}$
2.064	1.31	294	1793	460	0.403	75.6	−6.397	−0.04	1.60	0.74

**Table 8:** Parameters of the MJC-2 model.

*E* [GPa]	*v*		*ρ* [kg/m^3^]	*A* [MPa]	*B* [MPa]	*n*	*α*	*Q* [MPa]	$C_{1}$	$C_{2}$	C
80.4	0.3		7800	820.94	919.24	0.58	0.52	195.91	43.47	2.79	0.022
${\dot{\varepsilon }}_{0}$ [s^−1^]	*p*	*m*	$T_{0}$ [K]	$T_{m}$ (K)	$C_{p}$ [J/kg K]	$D_{1}$	$D_{2}$	$D_{3}$	$D_{4}$	$D_{5}$	$D_{6}$
4.2 × 10^−4^	2.064	1.31	294	1793	460	−0.567	3.437	−1.057	−0.04	1.60	0.74

**Table 9:** Comparison of experimental and numerical results.

*V_0_* [m/s]	Test *d_f_* [mm]	MJC *d_f_* [mm]	MJC-2 *d_f_* [mm]	Test *l_f_* [mm]	MJC *l_f_* [mm]	MJC-2 *l_f_* [mm]
231.6	8.82	8.36	8.51	26.43	25.59	26.18
257.3	9.29	8.76	8.95	25.71	26.28	25.45

## Data Availability

The original contributions presented in the study are included in the article; further inquiries can be directed to the corresponding author.

## Nomenclature

FE	Finite element
MJC	Modified Johnson–Cook
BLV	Ballistic limit velocity
SRB	Smooth round bar
NRB	Notch round bar
UTM	Universal testing machine
DIC	Digital image correlation
SHPB	Split Hopkinson pressure bar
GL	Gauge length
PEEQ	Equivalent plastic strain
F	Force
U	Displacement
U_d_	Diameter reduction
*u*_f_	Fracture displacement
$\varepsilon_{f}$	Fracture strain
$A_{0}$	Initial cross-sectional area
$d_{0}$	Initial diameter
$d_{f}$	Fracture diameter
*Ψ*	Reduction of area
σ	True stress
$\sigma_{u}$	Ultimate tensile strength
$\sigma_{e}$	Engineering stress
$\varepsilon_{e}$	Engineering strain
*t*	Time
$C_{0}$	Elastic wave velocity
$\varepsilon_{i}$	Strain caused by the incident wave
$\varepsilon_{r}$	Strain caused by the reflected wave
$\varepsilon_{t}$	Strain caused by the transmitted wave
$\varepsilon_{\mathrm{eq}}$	Equivalent plastic strain
${\dot{\varepsilon }}_{\mathrm{eq}}^{*}$	Dimensionless strain rate
$\dot{\varepsilon }$	Strain rate
*T^*^*	Dimensionless temperature
*E*	Modulus of elasticity
ν	Poisson’s ratio
*ρ*	Density
*A*	Yield stress
*α*	Strain hardening parameter
*B*	Strain hardening parameter
*n*	Strain hardening parameter
*Q*	Strain hardening parameter
$C_{1}$	Strain hardening parameter
$e_{0}$	Strain hardening parameter
$C_{1}$	Strain hardening parameter
$C_{2}$	Strain hardening parameter
*C*	Strain hardening parameter
${\dot{\varepsilon }}_{0}$	Reference strain rate
*p*	Thermal softening coefficients
*m*	Thermal softening coefficients
$T_{0}$ (K)	Room temperature
$T_{m}$ (K)	Melting temperature
$C_{p}$ (J/kg K)	Specific heat
η	Stress triaxiality
${\bar{\eta }}_{\mathrm{av}}$	Average stress triaxiality
$D_{1}-D_{5}$	Parameters of the MJC fracture criterion
$V_{0}$	Initial velocity
$V_{r}$	Residual velocity
$V_{bl}$	Ballistic limit velocity
